# Improving Quality and Access to Care in Long-Term Care Settings

**DOI:** 10.1093/geroni/igab046.1268

**Published:** 2021-12-17

**Authors:** Nancy Dudley, Nancy Dudley

## Abstract

Innovative delivery models that assure access to quality care in long-term care settings are needed for the diverse high-risk aging U.S. population. The 2008 National Academy of Medicine report, “Retooling for an Aging America: Building the Health Care Workforce” highlighted the need for changing the roles of health care providers in order to provide high-quality and cost-effective care to older Americans. Moreover, from the providers’ perspective, workplace violence in health care institutions, such as nursing homes, negatively affect the delivery, quality, and accessibility of health care. In this symposium, we identify needs and care provisions in the context of older adults aging in long-term care settings and discuss the implications for policy and health care transformation. This symposium comprises three distinct presentations: (1) identifying and addressing the needs of diverse older adults aging in low-income independent living facilities in community health practice; and based on pilot survey results from nurses in the State of New Hampshire, (2) reframing residents’ violence directed toward providers as self-protection and (3) proposing legislative and policy changes designed to meet the needs of staff and residents of long-term care facilities. These presentations represent efforts across long-term care settings to improve access and quality of care in the context of diverse older adults aging in the U.S.

